# Enhanced PDR-BLE Compensation Mechanism Based on HMM and AWCLA for Improving Indoor Localization

**DOI:** 10.3390/s21216972

**Published:** 2021-10-21

**Authors:** Harun Jamil, Faiza Qayyum, Faisal Jamil, Do-Hyeun Kim

**Affiliations:** 1 Department of Electronics Engineering, Jeju National University, Jejusi 63243, Korea; harunjamil@hotmail.com; 2 Department of Computer Engineering, Jeju National University, Jejusi 63243, Korea; faizaqayyum@jejunu.ac.kr (F.Q.); faisal@jejunu.ac.kr (F.J.)

**Keywords:** ePDR-BLE compensation mechanism (EPBCM), unscented Kalman filter (UKF), Kalman filter (KF), received signal strength indicator (RSSI), hidden Markov model (HMM), Bluetooth low energy (BLE)

## Abstract

This paper presents an enhanced PDR-BLE compensation mechanism for improving indoor localization, which is considerably resilient against variant uncertainties. The proposed method of ePDR-BLE compensation mechanism (EPBCM) takes advantage of the non-requirement of linearization of the system around its current state in an unscented Kalman filter (UKF) and Kalman filter (KF) in smoothing of received signal strength indicator (RSSI) values. In this paper, a fusion of conflicting information and the activity detection approach of an object in an indoor environment contemplates varying magnitude of accelerometer values based on the hidden Markov model (HMM). On the estimated orientation, the proposed approach remunerates the inadvertent body acceleration and magnetic distortion sensor data. Moreover, EPBCM can precisely calculate the velocity and position by reducing the position drift, which gives rise to a fault in zero-velocity and heading error. The developed EPBCM localization algorithm using Bluetooth low energy beacons (BLE) was applied and analyzed in an indoor environment. The experiments conducted in an indoor scenario shows the results of various activities performed by the object and achieves better orientation estimation, zero velocity measurements, and high position accuracy than other methods in the literature.

## 1. Introduction

Indoor location-based services (LBS) have always been of great importance because people live and work in indoor environments most of their lives. Massive wireless networks are built according to the IEEE 802.11 wireless Ethernet standard [1]. LBS are the backbone of indoor mobile positioning techniques [2]. Global navigation satellite systems (GNSS) and global positioning systems (GPS) have helped us provide accurate location services for outdoor environments. Still, these services are impeded by signal absorption and hamper in specific environments (e.g., dense forests, mountainous regions, underground facilities, and high-rise buildings) [3]. GNSS and GPS take the user′s three-dimensional information (i.e., longitude, altitude, and latitude) [4]. The accuracy of these services depends upon the line of sight, and if the precision is good, object detection is possible within meters. Therefore, to come up with an overall location service, a number of technologies have emerged for indoor positioning; for instance, radio frequency identification (RFI) [5], pseudo-satellites [6], indoor ultrasonic positioning (UIP) [7], indoor positioning approach using iBeacon [8], ultra-wideband (UWB) technology allows micro-positioning of objects along with obstacles [9], and indoor positioning based on ZigBee [10].

A large variety of reconfigured wireless devices (RWD) are already in use, namely Bluetooth low energy (BLE) beacons, smartphones, Wi-Fi, ultra-wideband (UWB) beacons, and digital cameras for indoor positioning [11,12]. For purpose-built installations, these RWDs are placed strategically throughout a defined space. IPS uses different localization mechanisms, including the position estimation of tagging devices from nearby anchor nodes, such as BLE, UWB, and Wi-Fi access points (AP) with known fixed positions. Weighted centroid localization (WCL) [13], distance vector hop (DV-Hop) localization [14], trilateration-based localization, context-recognition aided-based PDR localization [15], and fingerprinting-based localization techniques are also used [16]. In recent years, researchers have focused on related technologies and methods that provide high precision positioning services.

Pedestrian dead reckoning (PDR) is an independent approach, and its primary principle is to calculate the step length, determine walking direction, and step size by using inertial sensors (IS) (i.e., accelerometer, gyroscope, and geomagnetic meter) to calculate the moving target information and positions [17]. Bluetooth low energy beacon is pulled in for indoor localization technology because of low energy communication via the Bluetooth beacons and broadcasting low-energy Bluetooth signals in a given range. BLE beacons send out an ID number via BLE channels triggering a specific action relevant to the location approximately ten times every second [18]. The distance between BLE beacons and smartphones was computed based on the received signal strength indicator (RSSI) values. To estimate the position, we used the centroid localization algorithm (CLA) and weighted centroid localization algorithm (WCLA) proposed by [17,18,19], which uses the BLE beacon coordinations to estimate the position of the smartphone. The position accuracy using CLA is very low in WCLA when using weight to improve position accuracy. In AWCLA, location accuracy is increased, but still, the error accuracy is high. Different filters were used to get the precise output, e.g., complementary filter, low-pass filter, Butterworth filter, Kalman filter, extended Kalman filter, unscented Kalman filter, alpha–beta filter, Gaussian filter, etc. These filters are used in the literature as data fusion filters, responsible for removing noise from sensor values and producing an estimate of the system′s state by taking the average of the new measurement and the system′s predicted state using a weighted average. Furthermore, different machine learning-based models were also developed to estimate the position in an indoor environment. Still, due to the massive amount of sensing data, these systems cannot provide a real-time position estimation because of high computational requirements. Due to advanced and sophisticated machine learning techniques, it is therefore required to estimate the real-time position of an object in an indoor environment by using a lightweight, intelligent solution. Machine learning techniques have been utilized in many fields, such as healthcare, finance, irony detection, citation classification, effective waste management, energy consumption forecasting, boreholes data analysis, groundwater resource planning, and education, to name of a few [20,21,22,23,24,25].

In this study, EPBCM was developed to track the position of an object in an indoor environment. Quaternions and their conversion to Euler angles are explained. Smartphone-based inertial measurement unit (IMU) sensors were used for the data collection. A complementary filter was used to integrate the information of pitch, roll, and angular velocity to obtain orientation tracking. The state vector used for the estimation of orientation in UKF contains the prior gyros bias errors and Euler angle errors. The HMM-based activity detection approach was developed to recognize the various activities (running, walking, writing on a whiteboard, working on the computer, walking upstairs) performed by the smartphone user. The state transitions probability matrix and observation probability matrix were calculated based on the changing magnitudes of the accelerometer values. The compensation mechanism increases the position accuracy by reducing the drift error. KF was used for smoothing RSSI measurements obtained from the BLE beacon. The average weighted centroid localization algorithm (AWCLA) was used for the proximity calculation between the smartphone and fixed BLE beacon position. Various performance analyses were used to evaluate the significance of the proposed EPBCM based on HMM and AWCLA, such as clustering of raw data to know about the activities performed, comparison of position accuracy of PDR, BLE, and EPBCM, indoor localization visualization using IMU sensor data, and orientation estimation based on AHRS and UKF.

The notable contributions of the proposed study is as follows:

- Developed mechanism based on complementary filter to integrate roll, pitch, and angular velocity information to obtain orientation tracking information.
- Developed HMM-based activity detection approach to recognize the various performed activities.
- Smoothing of RSSI measurements by passing through the Kalman filter to remove noise and enhance the accuracy of distance calculation between the beacon and mobile phone user.
- Weight assignment based on the power of RSSI measurements and use of AWCLA for the proximity calculation between the BLE beacon and smartphone.
- Furthermore, different evaluation metrics were utilized to evaluate the effectiveness of the proposed EPBCM based on HMM and AWCLA, such as a comparison, in terms of position accuracy, confirmation of activity detection by clustering the sensor data to visualize the performed activities and compare with HMM-based activity detection approach, and comparison of the orientation estimation approach based on AHRS and UKF.

The rest of the paper is divided into the following sections. Section 2 presents the related works; Section 3 presents enhanced PDR-BLE compensation mechanism based on HMM and AWCLA for Improving indoor localization. Section 4 presents Compensation mechanism based on AWCLA. Section 6 discusses experimental design, implementation environment, comparison of proposed system with state-of-the-art-techniques, and performance analysis. Section 7 concludes the paper with possible future directions.

## 2. Related Work

The internet of things is an evolving field, attracting much attention from the research community [26,27,28,29,30,31,32,33,34,35,36,37,38,39,40,41,42,43]. The systems pertaining to location tracking and intelligent navigation are widely employed to handle specific scenarios related to security, logistic medicine, and mission-critical indoors. Indoor Localization is deemed one of the quintessential areas among all of them because of excessive location-based services [44]. Therefore, indoor positioning is a popular topic that is gaining popularity. It is a network of devices used to locate objects or people where global navigation satellite system (GNSS) and global positioning system (GPS) technologies lack exactness or are completely unsuccessful, particularly parking garages, underground locations, multistory buildings, railway stations, and airports. The dependency of multiple computing concepts on positionings, such as location and context-aware systems and ubiquitous computing, shows how important the indoor positioning systems (IPS) hold [45]. To find the accurate location where the GPS signal is lost, radio-frequency signals have been developed. When compared to the classic Bluetooth, Bluetooth low energy beacons are low in cost, low in energy consumption, and small in size [18]. Distance computation between beacons and smartphones can be done by using BLE beacons. To determine a single point in a two-dimensional space, a minimum of three beacons are required if considering the trilateration scenario [46]. Indirect distance computation involves the measurement of RSSI values.

A microelectromechanical systems (MEMS) are used to offer beacon-free solutions, consisting of a gyrometer, an accelerometer, and magnetometer. Due to the lightweight, smaller size, and lower cost of MEMS, sensors are often employed for the PDR system. Conventionally, the indirect estimation of walking speed and walking direction is conducted by using the PDR approach [47]. PDR can only obtain relative positioning results, has high position accuracy, and cumulative errors. However, PDR is a self-confined approach, but will produce a growing drift as the walking distance increases [48]. To reduce the errors, bias, and bias instability that accumulate through the navigation equation at the output of MEMS sensors can be contained by attaching inertial measurement sensors (IMU) on foot and other body parts to recognize particular activities and by using the zero-velocity update (ZVU) algorithm to predict the position [49,50]. The velocity and the acceleration integral of the foot are practically zero when the stance phase occurs during the motion of the pedestrian [51]. Therefore, an error in the foot orientation increases the position′s error to grow linearly with time. Similarly, the calculation of the non-gravitational acceleration is affected by the attitude errors. If the orientation and attitude errors are not tackled rightly, the combined result induces a worse position estimation. The erroneous step increased position error. Therefore, a low false detection rate, in estimating each step′s start and end is conducive to improve accuracy. In [52], for precise human body part orientation tracking, the author reviewed the filtering techniques and main sensor fusion. The author in [53] deals with the nonlinear attitude prediction system and linearization of the currently estimated points of the model in the PDR system by using the extended Kalman filter (EKF). For attitude estimation, the author in [54] proposed the hidden Markov model (HMM) based EKF. To enhance the results of the roll, pitch, and yaw for orientation tracking, the author developed a double-stage Kalman filter (KF) in [55]. In attitude estimation of PDR, when the nonlinear characteristic is extreme, for instance, environmental magnetic variation, variation in acceleration based on human motion, the attitude dynamics, etc., causes divergence of the EKF and degrades the accuracy. To enhance the performance of PDR by using a sophisticated ZVU algorithm in [56], the author achieved ∼4% distance error by incorporating the two outputs of the maximum bounds at the stance phase by implementing a binary decision-making rule. The study in [57] shows that the author knows the sensor data, based on sensor knowledge, examines the acceleration-magnitude detector, acceleration-moving variance detector, and the angular rate energy detector. The paper shows that all detectors can be derived by using the general likelihood ratio test (LRT) framework. To accurately distinguish stance phases, the author in [57] proposed a clustering algorithm. In [58,59], the authors suggest that HMM chains classify human activities, and [60] show that there are more computational costs for the hierarchically structured approaches. The author in [61] used a smartphone-based IMU sensor for activity recognition to facilitate the people who had chronic neurodegenerative diseases, such as Alzheimer′s.

To identify and track objects automatically, various technologies related to indoor positioning include Wi-Fi, ultra wide band (UWB), radio frequency identification (RFID), and BLE. To achieve better positioning accuracy and to reduce the positioning system cost based on RFID, the authors in [62] presented a hyperbolic positioning algorithm and in [63] convert paths that are non-line of sight (NLOS) to the paths which are line of sight (LOS) by employing a hybrid method based angel of arrival (AOA) and phase difference of arrival (PDOA). Positioning using BLE beacons in an indoor environment, the author suggested a hybrid method, combining dead reckoning and trilateration [64]. In [65], the authors suggested KF, to smooth the RSSI measurements obtained from the installed BLE beacons and developed an android application to locate a user inside a building. There were high computational and processing times in the UWB-PDR-based localization algorithm. Due to the increased numbers of anchors in the localization algorithm, the system cost also increased [66]. In [67], WCL and fingerprint techniques are used for indoor localization based on the least square method. Improvements are being made in the WCL localization algorithm by [68]. However, the estimated error is still significantly high.

To predict and measure the motion of the body based on IMU wearable devices, many machine learning algorithms are used. To reduce the error in an indoor environment, a deep neural network fingerprinting based indoor positioning approach is used [69]. In [70], the author used inertial sensor data to detect step length and step detection by using ANN and RNN. In another paper, the author used the feed-forward NN approach to get the location at the output [71]. Similarly, in [72], based on RSS measurements of Wi-Fi access point nodes, the author suggested a radial basis function to find the location of a user. As mentioned above in Table 1, the critical analysis of indoor localization based on the BLE-beacon, PDR machine learning algorithms, and the combined hybrid approach has many drawbacks in system performance and accuracy. To predict and identify the object or location, these approaches directly use sensor data as an input to the machine learning algorithm. The drifting error and bias in sensor readings affect the accuracy of the position estimation.

To the best of the authors′ knowledge, all of the indoor positioning systems are developed based on single or hybrid localization algorithms that have problems of high position errors and more computational costs, particularly for hybrid localization algorithms. Moreover, all of these only determine the location of an object in an indoor environment. In some cases, sensors mounted on the foot, hand, arm, and chest are considered to track the orientation and detect the activity of an object up to the binary level. Furthermore, all of these existing indoor positioning systems face different issues, such as high localization errors, high computational costs, and high hardware costs, to name of few, as mentioned in Table 1. Therefore, a new solution is required to track the position and detect the activities performed by the object, which aims to minimize latency, maximize position accuracy, and provide better orientation estimation. A compensation mechanism is developed to reduce the effect of the drift caused by the dead reckoning localization algorithm. Proximity calculation between beacons and smartphones are also improved by using AWCLA and passing RSSI measurements through the Kalman filter. This enhanced hybrid localization algorithm is presented in the following sections.

## 3. Enhanced PDR-BLE Compensation Mechanism Based on HMM and AWCLA for Improving Indoor Localization

### 3.1. Design of Proposed EPBCM Localization Algorithm

**Figure 1:**
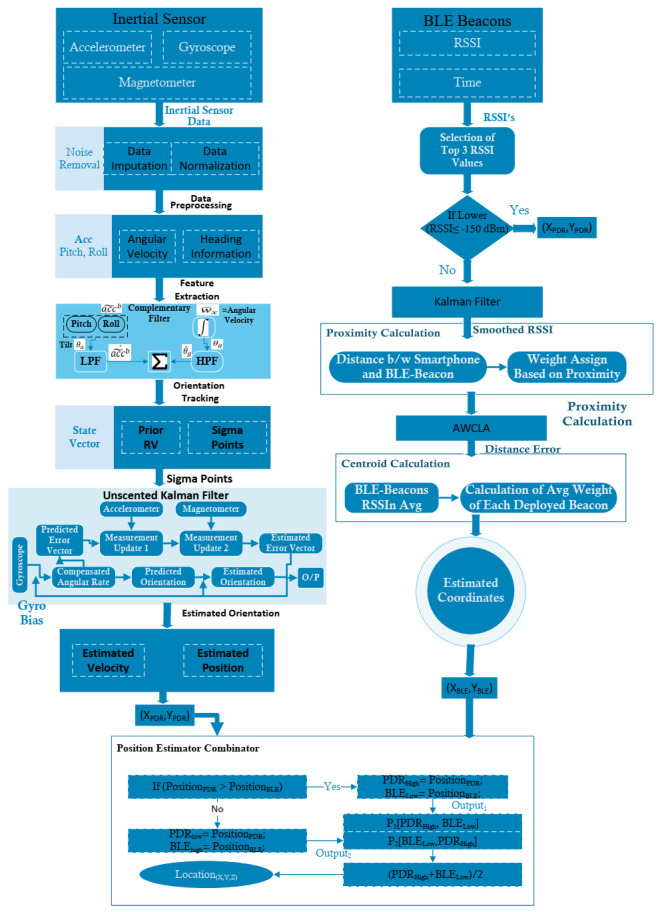
Hybrid position error compensation mechanism For indoor localization.

In this section, an EPBCM based on HMM and AWCLA was implemented to decrease drift and error in the position caused by navigation algorithms. The details of the proposed EPBCM are shown in Figure 1. A brief explanation of each step and mathematical formulation used to calculate the orientation, x,y coordinates through various localization techniques, and detection of activities of an object in an indoor environment by considering the HMM are presented in the below sub-sections. Table 2 presents notations and symbols used in mathematical formulation.

#### 3.1.1. Quaternions Calculation

The orientation between the inertial navigational frame {inf} and the sensor-body frame {sbf} can be represented by adopting quaternions. The scaler part of quaternions consist of s∈R and v∈(x,y,z), which is a vector part and are expressed as q≡(s,v), where $v\in R^{3}$. The vector part of the quaternions can be represented into two different frames *q* and $q^{*}$ as $v^{inf}=q⨂v^{sbf}⨂q^{*}$. The vector in the sensor body frame {sbf} refers to $v^{sbf}$ and the inertial navigational frame {inf} refers to vector $v^{inf}$, and ⨂ denotes the multiplication operations between vectors. A quaternion number is represented in the form of real and imaginary elements, where i,j, and *k* are basis elements and α,β,γ and δ are real numbers α+βi+γj+δk. The unit-vector quaternion $q_{inf}^{sbf}$ encoding rotation from the inertial frame to the body frame of the sensor $q_{inf}^{sbf}={\left(\alpha \;\sigma_{1}\;\sigma_{2}\;\sigma_{3}\right)}^{T}$.

The amount of the rotation that should be performed about the vector part specifies by the element α, where as elements $\sigma_{1}$, $\sigma_{2}$, and $\sigma_{3}$, thought of as a vector about which rotation should be performed. $q_{inf}^{sbf}={\left(\alpha \sigma^{T}\right)}^{T}$. If ϕ is the angle of rotation and the vector $\varepsilon ={\left(\varepsilon_{x}\;\varepsilon_{y}\;\varepsilon_{z}\right)}^{T}$ is a unit vector representing the axis of rotation, then the quaternion elements are defined as a unit quaternion ${\left\|q\right\|}^{2}=\alpha^{2}+\sigma_{1}^{2}+\sigma_{2}^{2}+\sigma_{3}^{2}=1$. A simple rotation can transform a vector from one reference frame to another according to Euler′s rotation theorem, and this is done by rotating ϕ about a unit vector ϵ. This unit vector representing the axis of rotation, and the quaternion elements are defined as:

(1) $$\left(\begin{array}{c} \alpha \\ \sigma_{1}\\ \sigma_{2}\\ \sigma_{3} \end{array}\right)=\left(\begin{array}{cc} \cos\frac{\varphi }{2}\\ \varepsilon_{x}\sin & \frac{\varphi }{2}\\ \varepsilon_{y}\sin & \frac{\varphi }{2}\\ \varepsilon_{z}\sin & \frac{\varphi }{2} \end{array}\right)$$

or as a rotation matrix $Q\left(q\right)=I+2\alpha S\left(\sigma \right)+2S^{2}\left(\sigma \right)$, S(σ) = $\left\lceil \begin{array}{ccc} 0 & -\sigma_{3} & \sigma_{2}\\ \sigma_{3} & 0 & -\sigma_{1}\\ -\sigma_{2} & \sigma_{1} & 0 \end{array}\right\rceil$, $Q\left(q\right)=(cos\frac{\phi }{2}$, ${\left(\varepsilon_{x}\;\varepsilon_{y}\;\varepsilon_{z}\right)}^{T}$×$sin\frac{\phi }{2}$. The set of quaternions, defined within a four-dimensional vector space over the real numbers $R^{4}$ is denoted by *Q*. Quaternions $q_{inf}^{sbf}$ can be used to rotate an arbitrary three-element vector from the inertial frame to the body frame using the matrix multiplication operation $v_{sbf}=Q_{inf}^{sbf}\left(q_{inf}^{sbf}\right)v_{inf}$. To rotate a vector from the inertial navigational frame to the sensor body frame, the attitude quaternion can be used to construct a 3×3 rotation matrix, to perform the rotation in a single matrix multiply operation expressed as:

(2) $$Q_{inf}^{sbf}\left(q_{i}^{b}\right)\;=\left[\begin{array}{ccc} \alpha^{2}+\sigma_{1}^{2}+\sigma_{2}^{2}+\sigma_{3}^{2} & 2\sigma_{1}\sigma_{2}-2\alpha \sigma_{3} & 2\sigma_{1}\sigma_{3}+2\alpha \sigma_{2}\\ 2\sigma_{1}\sigma_{2}+2\alpha \sigma_{3} & \alpha^{2}-\sigma_{1}^{2}+\sigma_{2}^{2}-\sigma_{3}^{2} & 2\sigma_{2}\sigma_{3}-2\alpha \sigma_{1}\\ 2\sigma_{1}\sigma_{3}-2\alpha \sigma_{2} & 2\sigma_{2}\sigma_{3}+2\alpha \sigma_{1} & \alpha^{2}-\sigma_{1}^{2}-\sigma_{2}^{2}+\sigma_{3}^{2} \end{array}\right]$$

The rotation can be reversed by simply inverting the attitude quaternion $q_{inf}^{sbf}$ before performing the rotation. Likewise, the operation can be reversed by negating the vector part of the quaternion vector. Euler angles are intuitive and straightforward for simple analysis and control, but these are limited by a gimbal lock, which prevents Euler angles from measuring orientation when the pitch angle approaches $\pm 90^{\circ }$.

#### 3.1.2. Quaternions Calculation at North–East Down (NED)

**Figure 2:**
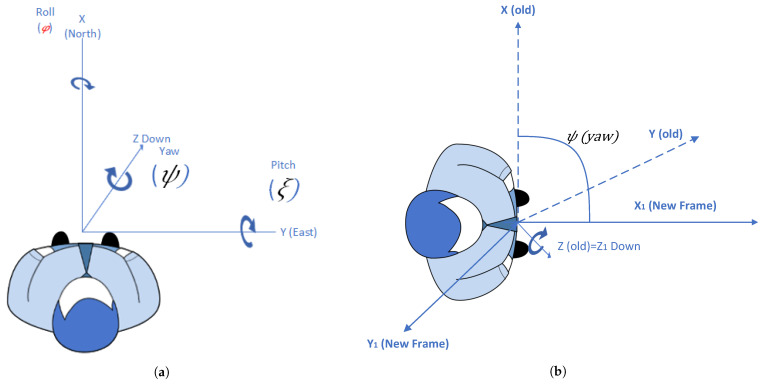
Quaternions calculation at north–east down (NED) reference. (**a**) Quaternions calculation at inertial frame; (**b**) Quaternions calculation at new frame.

The inertial frame is an unmoving Earth-fixed set of axes reference. In the inertial frame, x-axes point north, y-axes point east, and z-axes point down. This configuration of axes in the inertial frame reference is called north–east Down (NED). The sequence of rotations is used to represent a given orientation, which is the first yaw, then pitch, and finally roll. YAW rotation results in the new coordinate frame as illustrated in the Figure 2a. Yaw represents rotation about the inertial-frame z-axis by an angle ψ. The rotation of yaw around the z-axis produces a new coordinate frame where the z-axis is aligned with the inertial frame, and the yaw angle ψ rotates the x and y axes The orientation of the new coordinate frame after the rotation of yaw is shown in Figure 2b.

(3) $$Q_{inf}^{newf1}\left(\psi \right)=\left[\begin{array}{ccc} \cos(\psi ) & \sin(\psi ) & 0\\ -\sin(\psi ) & \cos(\psi ) & 0\\ 0 & 0 & 1 \end{array}\right]$$

Similarly, if the rotation around pitch represents ξ and the pitch is not rotating about the inertial frame Y-axis as shown in Figure 2, it is because that change in orientation occurs due to the changing in frames. This can be seen in Equations (4) and (5).

(4) $$Q_{\mathrm{newf}\;1}^{\mathrm{newf}2\;}\left(\xi \right)=\left[\begin{array}{ccc} \cos(\xi ) & 0 & -\sin(\xi )\\ 0 & 1 & 0\\ \sin(\xi ) & 0 & \cos(\xi ) \end{array}\right]$$

(5) $$Q_{inf}^{\mathrm{newf}\;1}\left(\xi ,\psi \right)=Q_{newf1}^{\mathrm{new}\;f2}\left(\xi \right)Q_{\inf\;}^{\mathrm{newf}\;}\left(\psi \right)$$

Performing rotation around the newf

2 x-axis, SBF is obtained. The SBF contains yaw, pitch, and roll, and the rotation matrix for moving from the newf2 to SBF is given by:

(6) $$Q_{\mathrm{newf}\;2}^{sbf}\left(\varphi \right)=\left[\begin{array}{ccc} 1 & 0 & 0\\ 0 & \cos(\varphi ) & \sin(\varphi )\\ 0 & -\sin(\varphi ) & \cos(\varphi ) \end{array}\right].$$

To complete the rotation matrix for moving from the inertial frame to the body frame—it is shown in Equation (7)

(7) $$Q_{inf}^{sbf}=\left(\varphi ,\xi ,\psi \right)=Q_{newf2}^{sbf}\left(\varphi \right)Q_{newf1}^{newf2}\left(\xi \right)Q_{inf}^{newf1}\left(\psi \right).$$

To get the accurate angular rates in the proper frames, the x-axis IMU output must be rotated into the newf2 frame, the y-axis IMU output must be rotated into the newf1 frame, and the z-axis IMU output must be turned into the INF. The resulting transformation matrix for converting body-frame angular rates to Euler angular rates is given by

(8) $$E=\left(\varphi ,\xi ,\psi \right)=\left[\begin{array}{ccc} 1 & \sin(\varphi )\tan(\xi ) & \cos(\varphi )\tan(\xi )\\ 0 & \cos(\varphi ) & -\sin(\varphi )\\ 0 & \frac{\sin(\varphi )}{\cos(\xi )} & \frac{\cos(\varphi )}{\cos(\xi )} \end{array}\right].$$

Let ρ represent the smartphone body frame x-axis gyro output, τ represent the smartphone body frame y-axis gyro output, and θ represent the body frame z-axis output. By taking the derivative of the roll, pitch, and yaw, the Euler angle rates are computed as

(9) $$\left(\begin{array}{c} \varphi \\ \xi \\ \psi \end{array}\right)=\left(\begin{array}{c} \rho +\tau \sin(\varphi )\tan(\xi )+\theta \cos(\varphi )\tan(\xi )\\ \tau \cos(\varphi )-\theta \sin(\varphi )\\ \tau \left(\frac{\sin(\varphi )}{\cos(\xi )}\right)+\theta \left(\frac{\cos(\varphi )}{\cos(\xi )}\right) \end{array}\right).$$

Gimbal lock becomes a problem when using Euler angles and the above operation illustrates mathematically. When the pitch angle ξ approaches $\pm 90^{\circ }$, the matrix elements diverge to infinity because the zero in the denominator causes the filter to fail.

The quaternions data can be converted to Euler angles on the receiving end, and the exact equations for converting from quaternions to Euler angles depend on the order of rotations. The sensors move from the inertial navigational frame to the sensor body frame using the first yaw, then pitch, and finally roll. The Euler angles can be obtained from the quaternions, and this results in the following conversion equation:

(10) $$\left[\begin{array}{c} \varphi \\ \xi \\ \psi \end{array}\right]=\left[\begin{array}{c} atan2\left(2\left(\alpha \sigma_{1}+\sigma_{2}\sigma_{3}\right),1-2\left(\sigma_{1}^{2}+\sigma_{2}^{2}\right)\right)\\ \mathrm{asin}\left(2\left(\alpha \sigma_{2}-\sigma_{3}\sigma_{1}\right)\right)\\ atan2\left(2\left(\alpha \sigma_{3}+\sigma_{1}\sigma_{2}\right),1-2\left(\sigma_{2}^{2}+\sigma_{3}^{2}\right)\right) \end{array}\right].$$

To tackle the problem that arose due to the arctan and arcsin, function atan2 computes the principal value of the argument function applied to the complex number in the quaternion. The function atan2 value is in the range (−π,π], that is −π<atan2(y,x)≤π. The body attitude matrix can be calculated by Euler angles φ, ξ, and ψ. By using the angular velocities of the body frame concerning the inertial navigation frame denoted in the smartphone body frame, the measure angular velocity can be calculated as explained in [48].

#### 3.1.3. Orientation Estimation Based on UKF

**Figure 3:**
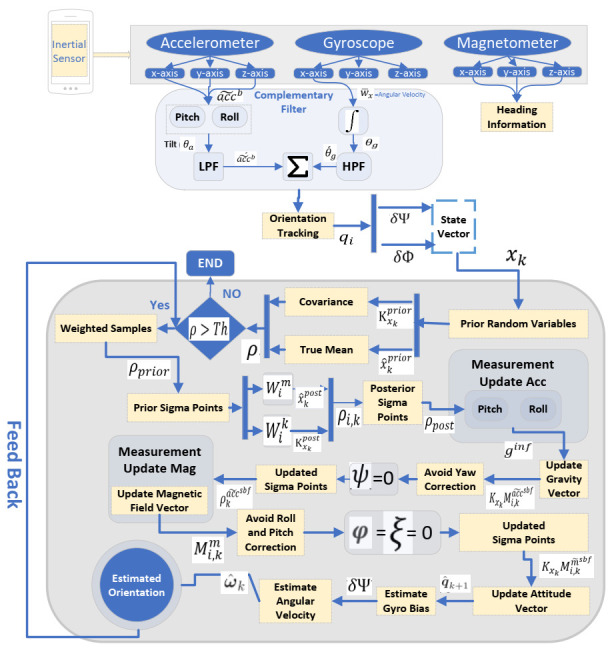
Orientation Estimation Based on UKF.

In this study, the detailed explanation of orientation estimation based on UKF is presented as shown in Figure 3. As discussed in Section 3.1.1, in the proposed orientation estimation based on UKF, the prior gyros bias errors, and Euler angle errors are expressed as δϕ and δψ in the state vector of the proposed filter as $x=\left[\begin{array}{c} \delta \psi \\ \delta \phi \end{array}\right]$. Assuming ‖δψ‖ is small and the error quaternions $e_{q}$ is approximated as $e_{q}≅\left[\begin{array}{cc} 1 & \frac{\delta \psi }{2} \end{array}\right]$ The basic idea behind the use of the proposed filter is to use the quaternions as the global attitude representation and use a three-component state vector δψ for the local representation of attitude errors [11].

(11) δΨ=δΨ.e

where δψ is the rotation error angle and $e^{t}=\left[\begin{array}{c} e_{x}\\ e_{y}\\ e_{z} \end{array}\right]$ is the rotation axis. The product of estimated and error quaternion gives true quaternion $q^{t}=\delta q\left(\delta \psi \right)⨂\hat{q}$. Where $e_{q}=\delta q\left(\delta \psi \right)$ and the cross symbol shows quaternion multiplication [68]. The state vector in the proposed filter is chosen as $\left[\begin{array}{c} \delta \psi^{T}\\ \delta \phi^{T} \end{array}\right]$, here δψ is the error between the estimated gyroscope bias and true gyroscope bias.

#### 3.1.4. Orientation Calculation System Model

The state equation for attitude estimation system is

(12) $$\begin{array}{cc} {\dot{x}}_{k} & =A_{k}x_{k}+Eu_{k}\\ x_{k} & ={\left[\begin{array}{cc} \delta \psi & \Delta \Phi \end{array}\right]}^{T} \end{array}$$

The process model is adopted as $x_{k+1}=\left(I+A_{k}\times \Delta t\right)\;x_{k}+u_{k}$, where $u_{k}$ is the noise vector, which refers to the noise related to the rotation error angle $\zeta_{\delta \Psi }\left(t\right)\;\mathrm{and}\;\zeta_{\Delta \Phi }\left(t\right)$ is the noise error between true bias random walk and estimated bias random walk. $A_{k}$ is defined using the estimated

(13) $$u_{k}=\left[\begin{array}{c} \zeta_{\delta \Psi }\left(t\right)\\ \zeta_{\Delta \Phi }\left(t\right) \end{array}\right],\;A_{k}=\left[\begin{array}{cc} -\left[{\hat{w}}_{b/n}^{b}\right] & -I_{3\times 3}\\ 0_{3\times 3} & 0_{3\times 3} \end{array}\right],\;\mathrm{and}\;E=\left[\begin{array}{cc} -I_{3\times 3} & 0_{3\times 3}\\ 0_{3\times 3} & I_{3\times 3} \end{array}\right]$$

rotation rate ${\hat{w}}_{b/n^{\prime }}^{b}\;\mathrm{and}\;\zeta_{\delta \Psi }\left(t\right)\epsilon R^{3}\;\mathrm{and}\;\zeta_{\Delta \Phi }\left(t\right)\in R^{3}$ are process noise and are assumed to be a zero mean Gaussian white noise. Then, the process covariance matrix is

(14) $$Q=E\left(u_{k}u_{k}^{T}\right)\;=\left[\begin{array}{cc} c_{\delta \Psi }^{2} & 0_{3\times 3}\\ 0_{3\times 3} & \zeta_{\Delta \Phi }^{2} \end{array}\right].$$

The measurement equation is represented by, $y=h\left(q\right)+{\left[{\tilde{acc}}^{sbf}\;{\tilde{mag}}^{sbf}\right]}^{T}$, where y is the measurement of the combination of accelerometer and magnetometer. Here ${\tilde{a\tilde{c}c}}^{b}\in R^{3}$ and ${\tilde{mag}}^{b}\in R^{3}$ are output of accelerometer and magnetometer, $\eta_{\mathrm{acc}{\;}^{sbf}}\;\mathrm{and}\;\eta_{\mathrm{magsbf}\;}$ are the measurement independent zero-mean Gaussian white-noise and can be expressed as the true magnetic and gravity vector $m^{n}$ and $g^{n}$.

(15) $${\tilde{acc}}^{sbf}=e_{q}^{*}⊗{\hat{q}}^{*}⊗-g^{inf}⊗\hat{q}⊗e_{q}+acc^{sbf}+\eta_{acc^{sbf}}$$

(16) $${\tilde{mag}}^{sbf}=e_{q}^{*}⊗{\hat{q}}^{*}⊗m^{inf}⊗\hat{q}⊗e_{q}+acc^{sbf}+\eta_{mag^{sbf}}$$

The variance of measurement noise is $\eta_{acc^{sbf}}^{2}\;\mathrm{and}\;\eta_{mag^{sbf}}^{2}$ and its covariance matrix expressed as $M_{R}=\left[\begin{array}{cc} \eta_{acc^{sbf}}^{2}\times I_{3\times 3} & 0_{3\times 3}\\ 0_{3\times 3} & \eta_{mag^{sbf}}^{2}.I_{3\times 3} \end{array}\right],$ and h(q) represents the nonlinear equations that convert the magnetometer reference vector $r_{m}ag\epsilon R^{3}$ and accelerometer reference vector $r_{a}cc\epsilon R^{3}$ from INF to the SBF. The values of $r_{a}cc$ and $r_{m}ag$ are constants whose specific values can be found using the method in [84].

#### 3.1.5. Time and Sigma Points Update

For calculating the posterior first and second order statistics of a random variable, which undergoes the unscented transformation. By using a minimal set of deterministically chosen weighted sample points, the state distribution is again represented by a Gaussian random variable (GRV). Covariance and true mean completely capture the prior random variable by using the weighted sample points called sigma-points. In this case, the error state *x* contains the orientation information *q* and the gyro bias ϕ and has zero mean $\hat{x}$. The following scheme is used to calculate the sigma points ρ.

(17) $$\rho_{j-1}=\left[{\hat{x}}_{j-1}^{2}+\lambda {\left(K_{j-1}\right)}^{\frac{1}{2}}{\hat{x}}_{j-1}-\lambda {\left(K_{j-1}\right)}^{\frac{1}{2}}\right]$$

where, λ represents the scaling parameter that shows the Sigma points spread around the column vectors of the covariance matrix *K* and $\hat{x}$.

(18) $$\lambda ={\left(\iota +\gamma \right)}^{\frac{1}{2}},\;\mathrm{and}\;\gamma =\mu \left(\iota +\kappa \right)-\iota$$

whereas, the augmented state vector dimension is represented by *l*, $\mu =(10^{-3},1],$ and K=0. To yield each point of a set, which is expressed through the Equation (52), and transformed samples $\rho_{i,j|j-1}=f\left(\rho_{i,j-1|j-1}\right).$ Here the *i*th column of the matrix represent *i*. The prior estimates of covariance $K_{x_{j}}^{\mathrm{prior}\;}$ and state ${\hat{x}}_{j}^{\mathrm{prior}\;}$ are given through the ρ as expressed in equation

(19) $${\hat{x}}_{j}^{prior}=\sum_{i=0}^{2l}W_{i}^{m}\left[\rho_{i,j|j-1}\right]$$

(20) $$K_{x_{j}}^{prior}=\sum_{i=0}^{2l}W_{i}^{j}{\left[\rho_{i,j|j-1}-{\hat{x}}_{j}^{prior}\right]}^{2}+Q_{j}.$$

In the above two equations $W_{i}^{m}$ and $W_{i}^{j}$ are used to calculate the mean and covariance of the posterior ρ′, respectively, as follows:

(21) $$W_{0}^{m}=\frac{\gamma }{{\left(l+\gamma \right)}^{\prime }},W_{0}^{j}=\frac{\gamma }{\left(l+\gamma \right)}+\left(1-\left(\mu^{2}+\beta \right)\right.$$

(22) $$W_{i}^{m}=\frac{1}{2(l+\gamma )},W_{i}^{j}=\frac{1}{2(l+\gamma )}$$

To determine posterior covariance, μ determines the spread of the ρ around ${\hat{x}}_{k}^{\mathrm{prior}\;}$, where β accentuate the weighting on the zero ρ [85]. The value of optimal β is obtained from [86].

#### 3.1.6. Measurement Update

Magnetic measurement and gravity vector are not good choices for the reckoning of the horizontal component of the state vector. Moreover, yaw correction is not possible by using a gravity vector. Due to the described issues and to ensure gravity correction, it does not act on the yaw estimate, and as magnetic anomalies only influence the pitch estimates and roll, we divide the measurement update into two steps. For the case of gravity vector measurement update:

(23) $$M_{i,j|j-1}^{\tilde{acc}sbf}=h_{a}\left(\rho_{i,j|j-1}\right)$$

We set the third part of δψ to zero after the first update step, as $q_{e,j}\left[x_{j}\right]=0$ and based on the update *K* the ρ should be recalculated. Similarly, the magnetic field vector measurement equation can be written as $M_{i,j|j-1}^{m}=h_{m}\left(\rho_{i,j|j-1}\right)$ and the first two part of δψ are set to zero after the second update as $q_{e,k}\left[2,3\right]=0.$ In UKF compared to EKF, the system around its current state does not need to linearize.

To adaptively adjust the measurement covariance *R*, diagonal covariance inflation (**CI**) approach in [87] is implemented. In this approach, for the residual error $(\tilde{y}),$ the measurement update of the low-confidence hypothesis will inflate in all directions. The proposed covariance inflation approach always guaranteed the Mahalanobis distance r<1. The CI approach can resolve the inconsistency between the low-confidence hypothesis observation and the proposed filter prediction. The measurement update for the accelerometer and magnetometer is

(24) $$\tilde{y}={\hat{q}}^{*}⊗g^{n}⊗\hat{q}-{\tilde{a}}^{b}$$

The determination of the error covariance $R_{k}$ is the main issue of implementing this method

(25) $$R_{k}^{a}=c\times {\tilde{y}}^{2}+\zeta_{a}^{2}$$

### 3.2. Activity Detection Model Based on HMM

The observation model uses the inertial measurement (IMU) acceleration data received from the different activities performed by the person containing the smartphone. By performing various activities while holding a smartphone in a user′s hand, fingerprints are collected of each activity in an offline phase. Let $\rho_{i,k,l}$ be the measurements, having set $M_{j}$ collected in the various designated areas of the 4th floor of JEJU national university, concerning the *k* activities performed ${\mathrm{PA}}_{k}$. Whereas $l\in \{0,1,2,\ldots ,M_{i-1}\}.$ Once new IMU measurements set $\rho_{t}$ collected during random motion, containing all of the mentioned activities in Table 3. The model allocates specific evidence to each zone.

In previous research, the observation model′s role was to assign a weight wO,t(.) to each activity performed in the various dedicated zones $Z_{i}\;i\in \left\{0,1,2,\ldots ,M(i-1\right)\}$ for all measurements in $\rho_{t}$. By modeling the activities based on the different values of the accelerations computed from various performed actions on the university floor, a distribution D(.) is constructed by the kernel density estimate.

(26) $$Dz_{i}\left(.\right)=\frac{1}{M_{i}}\sum_{l=0}^{M_{i-1}}\frac{1}{h_{i-1,1\ldots }h_{i-1},M_{PA}}\prod_{k=1}^{M_{PA}}K\left({}^{\times }-\rho_{i,k,l}/h_{i,k}\right),$$

To each PA in each zone K(.) is the kernel and $h_{i,k}$ is the bandwidth of the kernel. Due to the facility of analytical derivations, the Gaussian kernel is considered, and the shape of the kernel has no impact on the model [88].

(27) $$K\left(u\right)=\frac{1}{{\left(2\pi \right)}^{1/2}}e^{-1/2^{u^{2}}}$$

By maximizing the pseudo-likelihood leave-one-out cross-validation, the bandwidth of $h_{i,k}$ is estimated. $h_{i,k}={\mathrm{argmax}}_{h}NL_{i,k}\left(h\right)$ where $NL_{i,k}\left(h\right)$ is computed as,

(28) $$NL_{i,k}\left(h_{i,k}\right)=\frac{1}{M_{i}}\sum_{l=0}^{M_{i-1}}\log\left[\sum_{\tilde{l}\neq l}K\left(\frac{\rho_{i,k,l}-\rho_{i,k,l}}{h_{i,k}}\right)\right]-\log\left[\left(M_{i}-1\right)h_{i,k}\right].$$

The allocation of weight by the observation model to each performed activity at any time t is computed as the output of the kernel density estimate of each zone followed by a normalization phase,

(29) $$w0,t\left(Z_{i}\right)\;=\frac{Dz_{i}\left(\rho_{t}\right)}{\sum_{j=0}^{M_{z-1}}Dz_{i}\left(\rho_{t}\right)}.$$

In this activity detection approach, based on the hidden Markov model, the mobility model is constructed. To determine the confidence level that the sensor resides in each zone, the probability is assigned based on the measurements of the accelerometer and combined with the evidence given by the observation model. At first, we provide a general overview of the HMMs and the confidence-based zone estimation combined with the observation model.

#### 3.2.1. Hidden Markov Models

A probabilistic model that can be used to represent observations, and these sequence of observations can either be independent, time-dependent, continuous, or discrete [89]. Let the $S_{N}$ HMM model be denoted by H, and the total states $S=\{S_{1},S_{2},\ldots ,S_{N}\}$. The objective of the *N*th order HMM model is to determine the corresponding state sequence $S=\{S_{1},S_{2},\ldots ,S_{\alpha }\}$, whenever the sequence of length α and $R=\{R_{1},R_{1},\ldots ,R_{\alpha }\}$ is given. For each present state, the probability of arriving at the next state is designated by the transition probability and termed as matrix A. The actual states are hidden from the observer and determine the likelihood that each type of observation is in each state by using observable data and termed as emission probabilities forming matrix *B*. Probabilities of starting at different states in HMM are represented as *T*. Based on the prior knowledge, it can be either random, any vector generated, or uniform. Thus any HMM can be represented as H=(A,B,T). The transition probability from the state *a* to *b* can be denoted by $P_{ab}a,b\in \left\{1,2,\ldots ,S_{N}\right\}$ and forming the matrix A. The output probability distribution forming the matrix B, and can be represented as ${\overset{`}{P}}_{a}\left(R\right),a\in \left\{1,2,\ldots ,S_{N}\right\}$. The complete explanation is in [90].

#### 3.2.2. Activity Detection Approach Architecture

In an indoor environment, the objective is to detect a change in the sensor state from one zone to another in a specific period. To determine the likelihood that the sensor has followed some trajectory, we use the HMMs. Since the states are hidden $S=\{S_{1},S_{2},\ldots ,S_{\alpha }\}$, we can observe a sequence $R=\{R_{1},R_{1},\ldots ,R_{\alpha }\}$, corresponding to a vector of acceleration magnitude measurements at each state. To observe the sequence *R*, we are interested in determining the probability P(R|H). The probability obtained by the observation model is combined with P(R|H) to determine a confidence level of having the accelerometer sensor values residing in each zone. The transition between two zones $Z_{i}$ to $Z_{j}$ can be represented by $S_{N}$ state HMM $H_{i,j}$, whereas a set of HMMs denoted as $H_{i,j},i,j\in \left\{1,2,\ldots ,M_{z}\right\}$. In each transition region, the number of states chosen by the user is represented as $S_{N}$.

**Figure 4:**
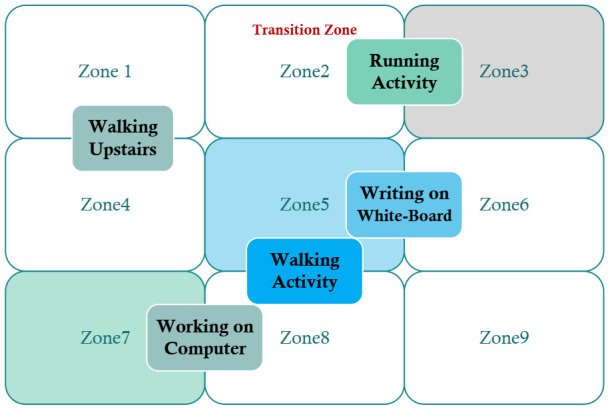
Transitions Regions between all performed Activities.

Figure 4 shows the transition region between each pair of neighboring zones in the offline phase. This region is divided into the number of states chosen by the user in each transition region. In each transition region, $\Gamma =S_{N}\times \sigma acc_{mag}$ measurements were gathered. Random selections of measurements from each state made the change in the frequency of the accelerometer values. Every kind of variation in the accelerometer values is considered as a database for each HMM. For each HMM $H_{i,j}=\left(A,B,\Gamma \right)$, the parameters are calculated as follows,

- Except for the first and the last state, where there are only two options, the accelerometer sensor values can move to the state upfront, behind, or retain their positions. The transition matrix $A_{S_{N}\times S_{N}}$.
- The activity detection model of each sequence is computed by modeling the offline collected accelerometer sensor measurements of each sequence with the multi-dimensional distribution $P_{a}\left(R\right)=Q_{\Gamma }\left(P_{a}\right),\;j\in \left\{1,2,\ldots ,M_{z}\right\}$. For example, to model observation ρa using the accelerometer sensor measurements Γ for all the PAs, the distribution $Q_{\Gamma }\left(\rho_{a}\right)$ which is the output of the distribution Q(.) is used.
- The vector T is defined as $\left[\begin{array}{ccc} \frac{1}{S_{N}} & ,..., & \frac{1}{S_{N}} \end{array}\right]$, unless information of prior knowledge is given regarding the starting of the state vector.

#### 3.2.3. Weight Assignment

The aim is to evaluate the probability of observing the sequence P(R|H), given an observation R and $S_{N}$ state HMM model *H*. When we know the parameters of the HMM, this is a problem for evaluating the observed sequence. For the evaluation purpose of P(R|H), it can be broken down as follows. We can compute the joint probability of the state sequence and the observed sequence, given a state sequence $S=\{S_{1},S_{2},\ldots ,S_{\alpha }\}$, and the range of α values in different zones are $\alpha_{1}\leq \alpha_{o}\geq \alpha_{2}$, $\alpha_{2}\leq \alpha_{o}\geq \alpha_{3}$, $\alpha_{3}\leq \alpha_{o}\geq \alpha_{4}$, $\alpha_{4}\leq \alpha_{o}\geq \alpha_{5}$, $\alpha_{5}\leq \alpha_{o}\geq \alpha_{6}$,

(30) P(R,S|H)=P(R|S,)×P(S|H)

In (30) is the product of the probability of the state sequence *S* given the model H and the likelihood of the observation sequence *R* given the state sequence *S*. The first term is obtained from the activity detection matrix **B** as follows,

(31) $$P\left(R_{\alpha i}|H\right)\;=\sum_{\alpha =1}^{\alpha_{i}}\prod_{a=1}^{\alpha i}{\overset{`}{P}}_{sa}\left(R_{a}\right)$$

From the transition matrix **A**, the second term is obtained as,

(32) $$P\left(S|H\right)=\prod_{a=1}^{\alpha i}P_{s_{a-1}s_{a}}\left(R_{a}\right)$$

By taking the summation of P(R,S|H) over all possible state sequences $S_{N}$, we can then derive $P\left(R|H\right)$ [91],

(33) $$P\left(R|H\right)=\sum_{\mathrm{For}\;\mathrm{all}\;S}P\left(R,S|H\right)=\sum_{\mathrm{For}\;\mathrm{all}\;S}\prod_{a=1}^{\alpha i}P_{s_{a-1}s_{a}}{\overset{`}{P}}_{sa}\left(R_{a}\right)$$

The computational cost and feasibility of the system depend on the range of α. To obtain the probability $P\left(R|H\right)$ and reduces the computational burden, a forward–backward algorithm can be used. Thus, (33) can be transformed as

(34) $$\begin{array}{c} P\left(R|H\right)=\sum_{y=1}^{S_{N}}P\left(R_{1},R_{2},\times ,R_{a},sa=P^{\prime }/H\right)\cdot P\left(R_{a+1},R_{a+2},\ldots ,R_{\alpha i}|,sa=\overset{`}{P},H)\right.\\ ga\left(\overset{`}{P}\right)=P\left(R_{1},\ldots ,R_{a},sa=\overset{`}{P}|H\right) \end{array}$$

(35) $$h_{a}\left(\overset{`}{P}\right)=P\left(R_{a+1},\ldots ,R_{\alpha }|sa=\overset{`}{P},H\right)$$

The probabilities $ga(\overset{`}{P})$ and $h_{a}\left(\overset{`}{P}\right)$ mentioned in (35) can be recursively computed; therefore, the probability $P\left(R|H\right)$ is given by,

(36) $$P\left(R|H\right)=\sum_{\overset{`}{P}}^{S_{N}}g_{a}\left(\overset{`}{P}\right)h_{a}\left(\overset{`}{P}\right).$$

In the (36), when a transition between $Z_{i}$ to $Z_{j}$ took place, the objective of the proposed HMM-based activity detection model is to assign evidence. Each HMM $H_{i,j}$ allocate a likelihood based on a sequence $R=\{R_{1},\;R_{1},\;\ldots ,\;R_{\alpha }\}$. For each HMM $H_{i,j}$, the probabilities $P\left(R|H_{i,j}\right),\;i,j\;\in \left\{1,2,\;\ldots ,\;M_{z}\right\}$ are computed. Based on that, the probabilities of the transitions between different zones are calculated. The probability is zero where no transition is possible. The coefficient of transition $c_{i,j},\;i,j\;\in \left\{1,2,\;\ldots ,\;M_{z}\right\}$ between zones $Z_{i}$ to $Z_{j}$ is as follows,

(37) $$c_{i,j}=\;\left\{\begin{array}{cc} P\left(R|H_{i,j}\right), & \;\mathrm{if}\;i\neq j\\ 1-\sum_{i=1}^{M_{Z}}P\left(R|H_{i,j}\right), & \;\mathrm{if}\;i=j. \end{array}\right.$$

Probabilities that the sensor moves from $Z_{i}$ to $Z_{j}$ are a complement to the probabilities where the likelihood for transition is zero. The calculation for the evidence $w_{M,t}\;\left(.\right)$ is as follows,

(38) $$w_{M,t}\left(Z_{i}\right)\;=\sum_{j=1}^{M_{z}}w_{O,t-1}\left(Z_{j}\right)\;\times \;c_{i,j}.$$

The weight associated with the observation model O(.) is $w_{O,t-1}$. The coefficient $c_{i,j}$ will be large if there is no transition $Z_{i}$ to $Z_{j}$. All coefficient $c_{i,j}$ values in the case for i=j will be significant. This problem does not cause any issue if we put the values of coefficients in the above equation and, thus, evidence is based on the observation model $w_{O,t-1}\;\left(Z_{j}\right)$.

#### 3.2.4. Zone-Based Confidence Estimation

The evidence was allocated by the mobility model and the observation model combined to get the confidence $C_{t}\left(.\right)$. The confidence level shows the zones based on the accelerometer sensor values.

(39) $$C_{t}\left(Z_{i}\right)\;=\frac{w_{0,t}\left(Z_{i}\right)\times w_{M,t}\left(Z_{i}\right)}{\sum_{x=1}^{M_{z}}w_{0,t}\left(Z_{x}\right)\times w_{M,t}\left(Z_{x}\right)}$$

Based on the accelerometer sensor values, the zone with the highest confidence level chosen is shown in (39).

### 3.3. Pedestrian Dead Reckoning

The zero-velocity information is effectual for the error correction because of the sensor drift and fast accumulation of the positioning error. The velocity and angular velocity are almost zero when the pedestrian′s foot is totally on the ground. For that purpose, the PDR system based on HMM and ZVU algorithms is introduced. According to the accelerometer and gyroscope readings, rules are defined based on the threshold for ZVU detectors [92]. This method ignores the sensor measurement fluctuations and uses an instantaneous sample that only gives the precise phase judgment. To recognize the phase, the following is used: $Obs=\;\left({obs}_{1},{obs}_{2},\;\ldots ,{obs}_{k}\right)$. To determine the observation ${obs}_{k}$ at *k* time, stance, and swing phase, two indicators are deployed. The accelerometer measurement is constant in the stance phase, the angular rate approximates to zero, and local gravity is approximately equal to the phase magnitude. Therefore, the observation is defined as ${obs}_{k}=Z_{1}\wedge Z_{2}$, whereas

(40) $$Z_{1}=\;\left|∥{\tilde{a}}^{b}∥-g\right|\;<TH_{1}$$

(41) $$Z_{2}=\;∥{\tilde{w}}^{b}∥\;<TH_{1}$$

where ${obs}_{k}=\;\left[0,1\right]$, ${TH}_{1}$ and ${TH}_{2}$ are thresholds. Thresholds are set empirically based on $Z_{1}$ and $Z_{2}$ calculated from the static test. To classify the observation into stance and swing, a long series of observations are given. To yield better recognition, the HMM is adopted, and to represent stance and swing, we use two HMMs, respectively. The calculation of the observations under each model and their conditional probability from the given observation sequence suggests the walking phase is the maximum probable model. For each HMM, the hidden state is assumed binary s=[0,1]. The output probability distribution *d*, which is $B=b_{j}\left(k\right)$ and state transition probability distribution $A=a_{ij}$ are the two elements.

(42) $$B=P(o_{t+1}=k|s_{t}=j)$$

and HMM is defined as ϵ=(A,B), and the parameters **A** and **B** are determined by using [57]. Partial observation sequence ${obs}_{1},{obs}_{2},\;\ldots ,{obs}_{t}$, ϵ being in state *i* at time *t*, and the forward probability is the probability of the HMM and it is shown in (43) as below,

(43) $$\mu_{t}\left(i\right)=P\left(obs_{1},obs_{2},\ldots ,obs_{T},|s_{t}=i,\epsilon \right)$$

From t+1 to the end, backward probability is the partial observation sequence probability, ${obs}_{t+1},{obs}_{t+2},\;\ldots ,{obs}_{T}$, given the state $s_{t}=i$ and the model threshold.

(44) $$\Phi_{t}\left(i\right)=P\left({obs}_{t+1},obs_{t+2},\ldots ,obs_{T}|s_{t}=i,\epsilon \right)$$

Given an observation history Obs and HMM $\epsilon^{n}$ to find new values of $\epsilon^{n+1}$, such that probability $P\left(Obs|\epsilon^{n+1}\right)\geq P\left(Obs|\epsilon^{n}\right)$. From the state *i* we estimate the expected number of transitions based on the changing values of accelerometer sensor values as $\sum_{t=1}^{T}\lambda_{t}\left(i\right)$ and $\sum_{t=1}^{T-1}\kappa^{\prime }\left(i,j\right)$ from state *i* to *j*. The complete derivation procedure to estimate the new model parameters in the last step can be found in [77]. The algorithm for the observation sequence—the maximum probable model as the walking phase having estimated parameters of HMM. $arg{}_{k}^{max}P\left(\epsilon_{k}|Obs\right)$ where k=0 or 1. Here, $\epsilon_{1}$ represents the stance model, and $\epsilon_{0}$ represents the swing model. By using the following equation during the swing phase, the velocity $v^{n}$ and the position $p^{n}$ are estimated by using the HMM-based ZVU algorithm.

(45) $$acc_{k}^{n}={\hat{q}}_{k}⊗\tilde{acc}c_{k}^{b}⊗{\hat{q}}_{k}^{*}-g^{n}$$

(46) $$\;\mathrm{velocity}{\;}_{k}^{n}=\;\mathrm{velocity}{\;}_{k-1}^{n}+acc_{k}^{n}\Delta t$$

(47) $$\;\mathrm{position}{\;}_{k}^{n}=\;\mathrm{position}{\;}_{k-1}^{n}+\;\mathrm{velocity}{\;}_{k-1}^{n}\Delta t+\frac{1}{2}acc_{k}^{n}\Delta t^{2}$$

The sampling interval of the inertial sensors is △t and $g^{n}$ and the gravity vector in inertial navigational frame {inf} is $g_{n}$.

## 4. Compensation Mechanism Based on AWCLA

In the case for the compensation mechanism based on AWCLA, we used iBeacon Estimote version Bluetooth 4.0 smart as a fixed device, and its detail is mentioned in Table 4. A 32-bit ARM® Cortex M0 is a small computer accompanied by a temperature sensor and accelerometer. Bluetooth low energy beacon uses ultra-high 2.4 GHz radio frequency. The CR2477 battery power source is used to power the BLE estimate for three years if used in the default condition. The range of the ideal Beacon is around 70 m, and in an indoor environment where signals can interfere, diffracted, or be absorbed by the walls and human body, the range is reduced to 30–40 m. The iBeacon sent information in an advertisement packet containing RSSI values, advertising intervals, measured power, and broadcasting power.

### 4.1. Kalman Filter Based RSSI Measurements Filtering

**Figure 5:**
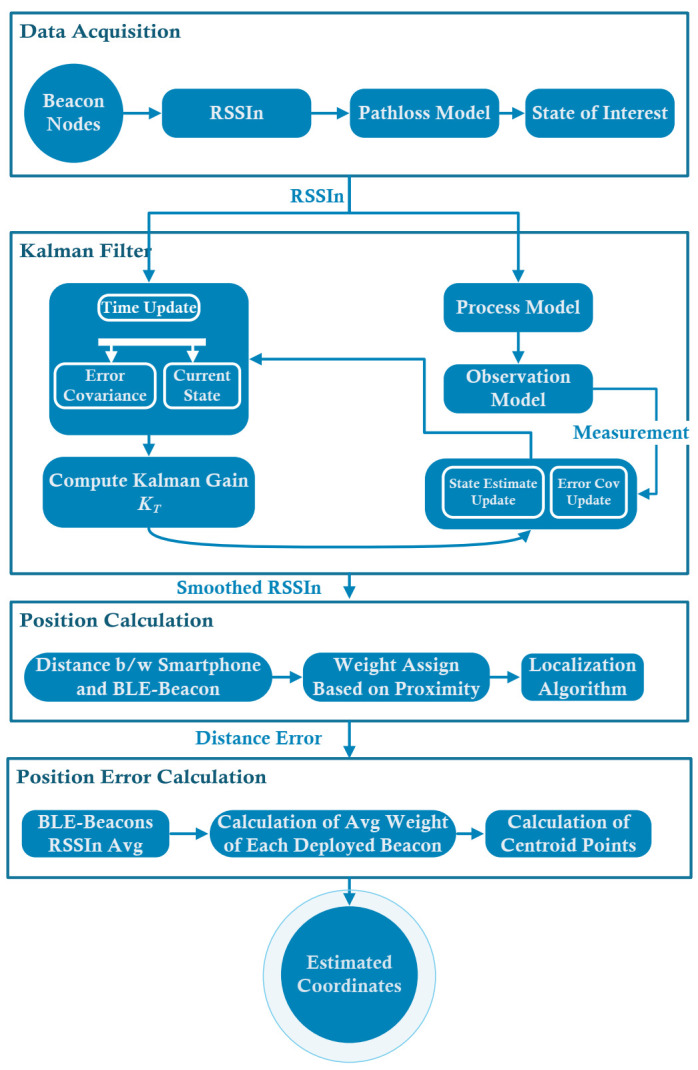
BLE-beacon Compensation Mechanism Based on Kalman Filter and AWCLA.

Figure 5 shows the distance computation between the object and Bluetooth low-energy beacons, which is used for calculating the position of an indoor object. Received signals from the beacons are used for the calculation of distance. To smooth RSSI signals, we used the proposed KF process and measurement model. The distance computing model and approach for RSSI estimation are presented. The distance output from iBeacons and the received RSSI measurements have suffered from high distance errors and high noise levels in the indoor environment. Various localization algorithms have been proposed to decrease the position error and enhance the accuracy when using the distance values based on the beacon output signals. However, still, the error is too high, and the localization accuracy is low. In this paper, indoor positioning accuracy and the distance error were improved using the proposed enhanced PDR-BLE compensation mechanism based on HMM and AWCLA for improving indoor localization.

### 4.2. Path-Loss Model-Based Measuring Distance

The nature of the medium and the distance are factors that cause an attenuation when wireless signals are transmitted. The signal is reflected, diffracted, refracted, and scattered when experiencing objects during transmission. In the case of LOS or NLOS, direct attenuated signal is due to other physical effects and indirect attenuated signals such as refraction, reflection, scattering, and diffraction [93]. In the simplest path loss model, there is no multi-path components and signal strength decreases by $\frac{1}{d1}$.The amount of power transmitted compared to the power received is calculated using Friis free space equation [94].

(48) $$\;\mathrm{received}{\;}_{P}=\;\mathrm{transmitted}{\;}_{P}\frac{Tx_{G}Rx_{G}{\overset{`}{\lambda }}^{2}}{{\left(4\pi D\right)}^{2}}.$$

The path loss is expressed in dB and it takes place exponentially with distance as shown in Equation (49)

(49) $$P_{loss}\left(d\right)\;=P_{loss}\left(d_{0}\right)\;+\;10nlog\left(\frac{d}{d_{0}}\right).$$

The relation between distance and RSSI in [80] can be described by the log-distance path loss model.

(50) $$RSSI=-10nlog10\left(\frac{d}{d_{0}}\right)+r_{0}$$

The above equation can be rewritten as:

(51) $$d=10^{\left(\frac{\left({Tx}_{s}-RSSI\right)}{10n}\right)}$$

where, the strength of the transmitted signal is ${Tx}_{s}$. The process model for smoothing RSSI using the Kalman Filter is shown below in Equation (52):

(52) $$x_{t+1}=Ax_{t-1}+Bu_{t}+w_{t}$$

The relationship is modeled through matrices **A** and **B**, and it is among the control unit, current state, and previous state. At time step t, the state of interest is $x_{t+1}$, whereas the previous state is $x_{t-1}$, $w_{t}$ is the process noise, and the control input is $u_{t}$. The KF based observation model is expressed as:

(53) $$m_{t}=TX_{t}+v_{t}$$

where, at time step t $m_{t}$ is the measurement, the transformation matrix is represented as **T**, and noise measurement is $v_{t}$.

Likewise, the steps update and prediction based on the Kalman filter are as follows

(54) $${\hat{x}}_{t}={\hat{x}}_{t-1}+Bu_{t}\;\left(State\;prediction\right)$$

(55) $${\hat{P}}_{t}=AP_{t-1}A^{T}+Q\;\;\;\left(Error\;Covariance\right)$$

(56) $$G_{K}=P_{t}^{-}T^{T}{\left(TP_{t}^{-}T^{T}+K\right)}^{-1}\;(\mathrm{Gain}\;\mathrm{Calculation})\;$$

(57) $${\hat{x}}_{t}={\hat{x}}_{t}^{-}+G_{t}\left(m_{t}-G_{t}T\right)P_{t}^{-}\;(\mathrm{Estimate}\;\mathrm{update})\;$$

(58) $$P_{t}=\left(I-G_{t}T\right)P_{t}^{-}\;(\mathrm{Error}\;\mathrm{covariance}\;\mathrm{update})\;$$

We define RSSI signals as the state of interest $x_{t}$. For certain time frames, the mobile and position are set as static; hence, the RSSI measurements in that time frame remain constant, and the rest of the parameters are taken as process noise. The model can be constructed by setting A to an identity matrix and ignoring control input $u_{t}$.

The RSSI estimation is computed in three steps. Step 1: $x_{t}=RSSI\left(t\right)$. Step 2: ${\dot{x}}_{t}=Ax_{t}+w_{t}$ (Kalman filter process model). Step 3: ${rssi}_{m}=Tx_{t}+V_{t}$, observation are designed by using the received RSSI measurements and the relationship between the state of interest. For the state of interest updates and the KF, we use a time step from t−1 to t, variance from the time step t−1 to *t*, and Kalman Gain (KG).

### 4.3. Position Estimation Using Beacon Weights

To find the mobile position the centroid location algorithm uses, while using the weight of each Beacon, the weighted centroid location WCL estimates the mobile location. In this paper, we explored the Beacon′s importance in terms of weight to estimate the mobile position based on CLA by using the deployed beacons. The accuracy of the centroid localization algorithm is poor, so different modifications are proposed to decrease the position error. To improve the object′s position, the proximity between the mobile devices and the Beacons are considered in terms of weights for each beacon. The relationship between the weight and distance is directly proportional, while the impacts of propinquity between the mobile devices and the beacons are inversely proportional. Based on (59), the value of Beacon weight is calculated using equation

(59) $$w_{ij}=\frac{1}{{({\hat{d}}_{ij})}^{g}},$$

where the estimated distance between the mobile device and beacon is denoted by $d_{ij}$ and the adjustable degree depends on the environments, and it is referred to *g*. To estimate the unknown mobile position, the weighted centroid localization algorithm uses Equations (60) and (61) based on the known Beacon position.

(60) $$\hat{x}=\frac{\sum_{i=1}^{n}w_{i}\times x_{i}}{\sum_{i=1}^{n}w_{i}}$$

(61) $$\hat{y}=\frac{\sum_{i=1}^{n}w_{i}\times x_{i}}{\sum_{i=1}^{n}w_{i}}$$

Here $\hat{x}$, $\hat{y}$ are the estimated x and y coordinate. The weight of each Beacon calculates using the signal power per Equation (62)

(62) $$w_{ij}={\left(ref\left(x,y\right)\;\times \;10^{\left(\frac{RSSI}{20}\right)}\right)}^{g}$$

Calculations of smartphone locations in indoor surroundings based on KF, which integrates the smoothed RSSI measurements based on the beacon weight. In the proposed algorithm based on KF, RSSI measurements are pre-processed and integrated. The proposed beacon-based localization eliminates noise and smooth RSSI values. By using the estimated filtered RSSI values, the distance between the deployed beacons and smartphones is calculated. The strength of the received RSSI signal is directly proportional to the power delivered to the beacon. The calculation of the actual distance between the deployed beacons and smartphones by using centroid points of beacons.

(63) $$d_{actual_{n}}={(\left(x_{n}-x_{0}\right)}^{2}{+\;{\left(y_{n}-y_{0}\right)}^{2})}^{\frac{1}{2}}\;\;\;\left(n=1,2,\ldots ,m\right)$$

The error in distance is calculated using equation $△d_{n}=d_{n}-dactual_{n}\left(n=1,2,\ldots ,m\right)$. The position error between the BLE beacon and smartphone is denoted by △dn. The estimated smartphone coordinates are calculated using weights of BLE beacons, and the average of the BLE beacons used to calculate the position. The estimated position of the smartphone is calculated as follows.

(64) $${\hat{x}}_{est}=\frac{\sum_{n=1}^{m}wn_{avg}\times \;\left(xc_{ij}\right)}{\sum_{n=1}^{m}wn_{avg}}\;\left(i\&j=a,b,c\right)\;\mathrm{and}\;i\neq j$$

(65) $${\hat{y}}_{est}=\frac{\sum_{n=1}^{m}wn_{avg}\times \;\left(yc_{ij}\right)}{\sum_{n=1}^{m}wn_{avg}}\;\left(i\&j=a,b,c\right)\;\mathrm{and}\;i\neq j$$

(66) $$E_{p}={\left({\left({\hat{x}}_{est}-x_{0}\right)}^{2}+{\left({\hat{y}}_{est}-y_{0}\right)}^{2}\right)}^{1/2}$$

**Figure 6:**
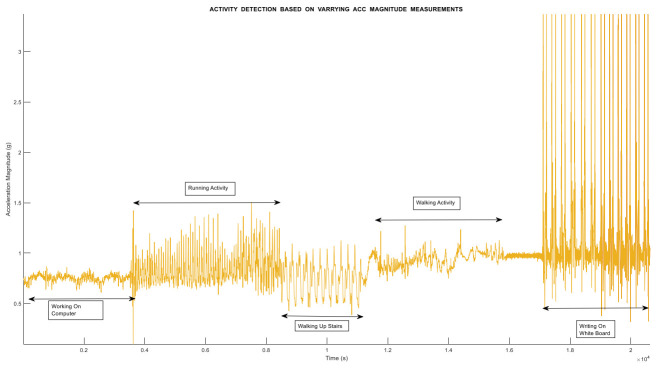
Activity detection using measurements of accelerometer sensor data.

**Figure 7:**
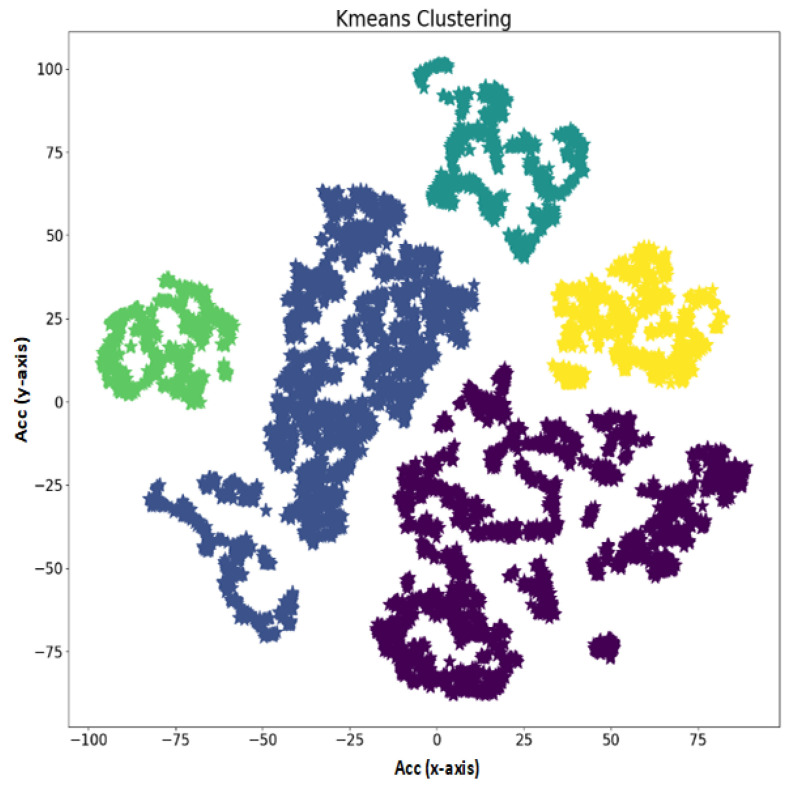
Clustering of IMU sensor data.

Figure 6 and Figure 7 shows the activities performed by an object in an indoor environment. In Figure 6, various performed activities are shown, which include working on a computer or idle activity, running activity, walking upstairs, walking on a plain surface, and writing on board. The change in the magnitude of accelerometer sensor values clearly illustrates the activities start and end times. It also shows how the change in the frequency also changes as the change in activity occurs. The high magnitude and high frequency in activity writing onboard show the frequent movement of the test object in an indoor environment. On the other hand, Figure 7 demonstrates the raw data information obtained from the IMU smartphone-based sensor.

## 5. Experimental Results and Discussion

### Development Environment

Experiments were conducted on a Windows PC with 12GB RAM. A front end (desktop application) was developed using Java, and the clustering techniques were applied in python. Well-know python libraries, including NumPy, SkLearn, and Scipy, were used for clustering experiments. In addition, NCSS was used for the visualization of data in PC. Furthermore, the simulation time for acquiring data for every instance was one minute (60 s). The required software and hardware components are listed in Table 5.

## 6. Results and Discussion

To validate our enhanced PDR-BLE compensation mechanism based on HMM and AWCLA for improving indoor localization, the person moves in an indoor environment with a Bluetooth enabled smartphone. The mobile was tested in seven different locations. Actual smartphone coordinates were compared with the estimated positions. Seven Bluetooth low energy beacons with known coordinates named $C_{1}\left(x_{1},y_{1}\right),C_{2}\left(x_{2},y_{2}\right),\ldots C_{n}\left(xn,yn\right)$(n−1,2,…6) placed in the entry and exit points of the fourth floor of Jeju National University, South Korea. To validate our approach, at eleven different locations, the position of the smartphone was calculated. The RSSI values of the BLE-beacon, as shown in the figure, were calculated in the android application. The positioning system in this paper is three-dimensional. In this paper, we considered two floors of Jeju National University engineering building 4. We used a 3D navigation approach to localize object movement in an indoor environment.

By using the Kalman filter in the proposed algorithm, the collected RSSI values were then smoothed, and for position estimation of the smartphone, the weight of each BLE-beacon was used. The figure shows various smartphone and deployed BLE beacon positions.

### 6.1. Error Reduction Using Kalman Filter in RSSI Measurement

**Figure 8:**
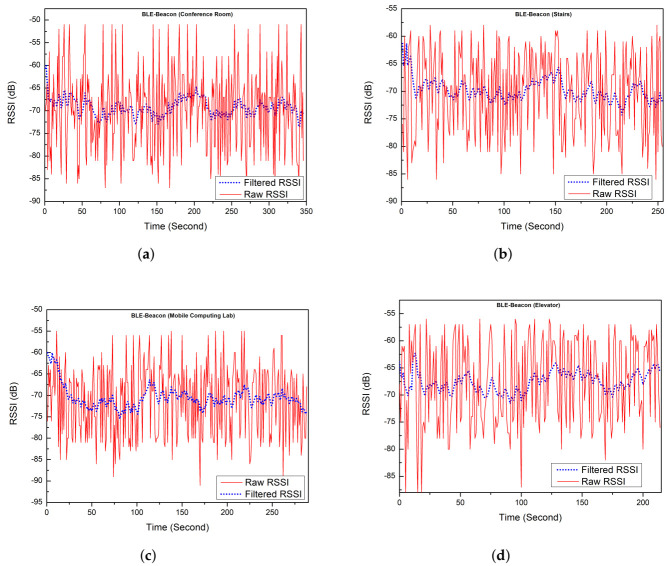
Filtered RSSI values and raw RSSI values. (**a**) BLE-beacon RSSI values around Conference room; (**b**) BLE-beacon RSSI values around stairs area; (**c**) BLE-beacon RSSI values around MCL; (**d**) BLE-beacon values around elevator area.

To validate our KF approach mentioned in Section 4, RSSI measurements passed through KF to reduce errors. The result of smoothed RSSI values obtained after passing raw RSSI measurements through the Kalman filter from different smartphone locations is mentioned in Table 6. On separate entry and exit points of the floor, the estimated BLE-beacon RSSI values and raw BLE-beacon RSSI values are shown in Figure 8. The smoothed BLE-beacon RSSI values are further used to calculate the distance.

#### 6.1.1. Comparison between BLE-Beacon, PDR and EPBCM Localization Algorithm

**Figure 9:**
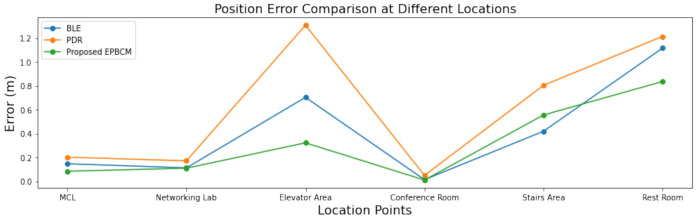
Position Calculation based on EPBCM, PDR, and BLE-beacon at different mobile locations.

The estimated mobile positions using EPBCM algorithm, the BLE-beacon and PDR is given in Table 7. The combined plotting of EPBCM, PDR, and BLE-beacon-based localization at all tested mobile positions is shown in Figure 9. The EPBCM algorithm shows promising results when compared with other IPS, such as PDR and BLE-beacon-based systems.

#### 6.1.2. EPBCM Algorithm Based Positioning

**Figure 10:**
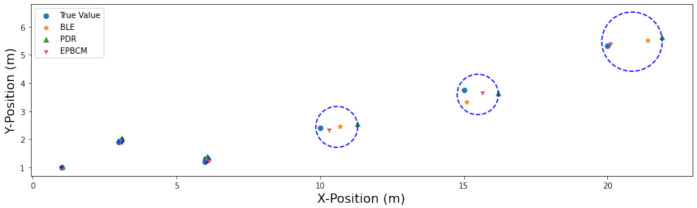
Mobile position estimation using BLE-beacon, PDR, and the EPBCM algorithm.

To validate the EPBCM localization algorithm, six mobile positions were chosen and compared with other estimated positions using different approaches. Figure 10 shows the actual position at six random mobile positions compared to their estimated location using EPBCM, PDR, and the BLE-beacon algorithm. The deviation of position obtained from the proposed EPBCM approach from the actual position is shown in Table 6, along with the coordinates of these locations and their errors.

**Figure 11:**
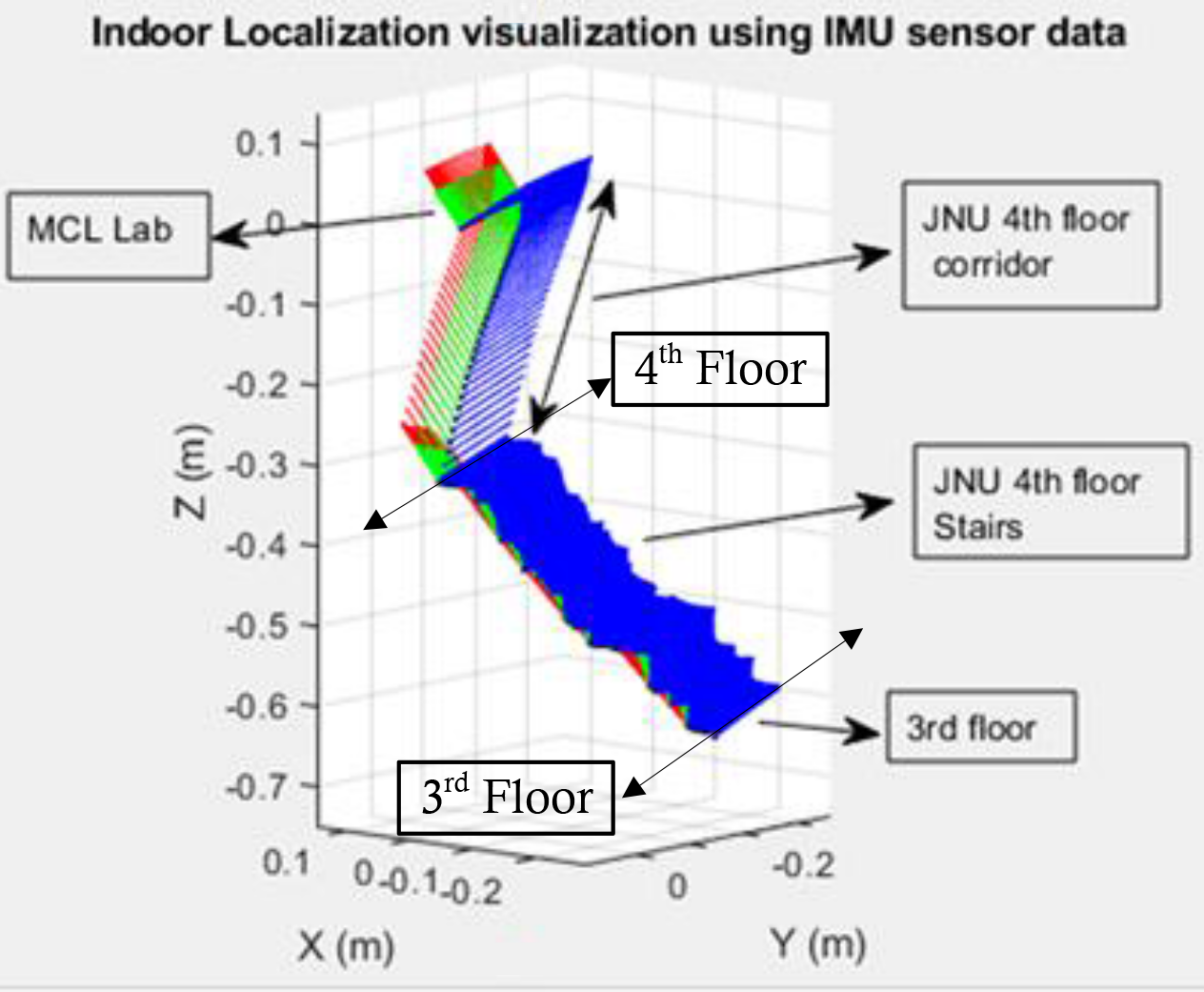
Indoor localization visualization using IMU sensor data.

The measurements of accelerometer, magnetometer, and gyroscope were used to track the position and orientation of an object moving in an indoor environment. Walking down two flights of stairs, trajectory is shown in Figure 11, whereas the count of steps in each flight is 13 steps.

The purpose of showing this result is to evaluate the performance of UKF-based orientation tracking and data collection from smartphone-based IMU sensors. The starting point is the mobile computing lab (MCL) situated on the fourth floor of the JNU, and the ending point is the end of two flights of stairs. However, the exact ground truth cannot be given, but the person′s orientation and activity can be determined. Compared to the flat ground, the error in stair walking is more significant. This is due to the fact that the calculations of vertical acceleration in stair walking has to be calculated for this scenario and it must include gravitational acceleration.

**Figure 12:**
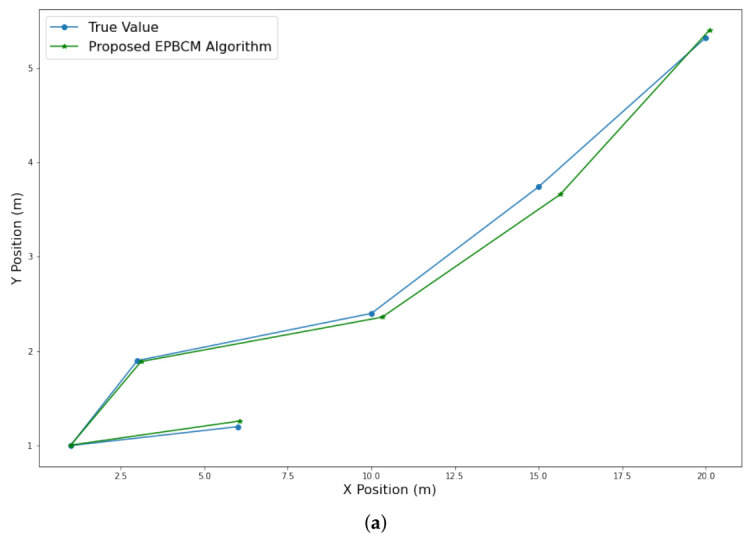

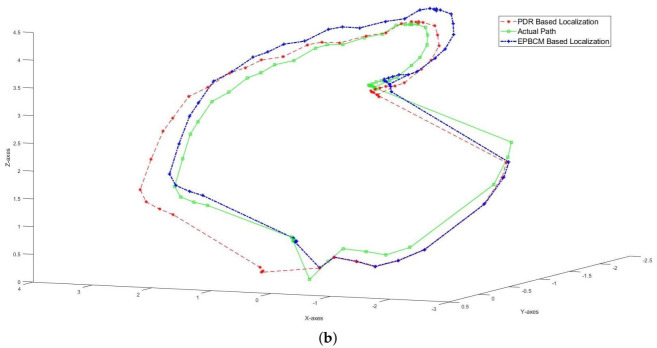
Performance evaluation of the proposed localization model considering pedestrian walking trajectories. (**a**) Pedestrian walking experiment; (**b**) Person walking trajectory evaluation by using EPBCM and PDR based localization algorithms.

In this experiment, several pedestrian walking experiments were conducted in the engineering building of JNU, and the final results are shown in Figure 12. In addition, walking experiments were conducted to assess the performance further, and the smartphone′s position was compared with the various reference points at the path of the test subject. Figure 12a shows pedestrian walking experiment conducted on the fourth floor of Jeju National University. It is clearly seen that the localization trajectory based on the proposed EPBCM algorithm follows the ground truth values. Figure 12b shows the complete round trip foot trajectory of walking along a corridor, walking down two flights of stairs, containing multiple turns, and the total distance covered is around 220.35 m. The trajectory computed with the proposed algorithm is compared with the PDR localization algorithm and actual trajectory. The proposed localization algorithm shows a clear decrease in the drift from the actual path as compared to the PDR. To quantify the positioning accuracy, we adopted the maximum error and end-to-end error. For the evaluation of the proposed algorithm, we implemented three algorithms EPBCM, PDR, and BLE. EPBCM incorporates accelerometer magnitude HMM-based detection, and the other two are PDR and BLE-beacon-based localization algorithms.

## 7. Conclusions

In this study, an EPBCM based localization algorithm was developed to estimate the position of an object in an indoor environment. The proposed system is a combination of two localization algorithms—PDR and BLE-beacons. In the proposed position estimation module, orientation is estimated using the unscented Kalman filter and HMM-based activity detection by considering the varying measurements of the accelerometer sensor. In this paper, the BLE-beacon-based localization algorithm is used as a compensation algorithm. In this compensation model, the path-loss model is used for distance measurement. The Kalman filter is used for noise elimination, smoothing RSSI measurements, and integrates raw BLE-beacons measurements to reduce the positioning error. The position estimation combinator is designed to combine the position coordinates of the two localization algorithms and get the enhanced position, containing less error. For comparative analysis, we compare the HMM-based activity detection approach results with the K-mean clustering technique. We also compare the three localization algorithm results, showing the proposed indoor positioning system containing the least error. It also shows that the proposed algorithm deviation from the actual location is the minimum. The walking scenario experimental results show that the proposed EPBCM based on HMM and AWCLA is feasible for the indoor positioning system. At some points, the positioning error is tiny, although to maintain a high accuracy level, more beacon deployment must be required to get more compensation in error at different mobile locations.

## Figures and Tables

**Table 1:** Critical analysis of existing technologies for positioning error in IPS.

Sensors	Technique	Environment	Max Distance	Error in Meters	Achieved Accuracy
Gyro, Acc [73]	Zero velocity update, map matching	Sensor mounted on person′s waist	40 m	0.683 m	98.26%
Mag, Acc [74]	PDR, map matching	Sensor in person′s pocket	104 m	(0.55–0.93) m	Ave LE, (0.55–0.93) m
Acc, Gyro [75]	Quaternion complementary filter	Smartphone placed in trousers, jacket, and held in hand	270 m	0.529 m	Above 98%
IMU [76]	Learning prediction system and improving parameters of the alpha–beta filter	NGIMU sensor attached to person′s body	∼50 m	0.102 m	Above 98.7%
IMU [77]	Learning module, based on ANN and KF are used as the prediction algorithm	Prediction of actual sensor reading from Noisy measurements	∼50 m	0.009 m	Above 99%
Acc, Gyro [78]	Model classification	Mobile phone in person′s hand and pocket while walking	168.55 m	0.31 m	Ave LE, 1.35 m
Acc, Gyro, Wi-Fi [56]	Zigbee RSSI fusion based on EKF with PDR	Zigbee and IMU sensor mounted on person waist	25 m	N/A	Max LE, 4 m
Acc, Gyro, Mag, RFI [79]	RFID RSSI fusion based on EKF with PDR	IMU mounted on person′s foot and RFID tags installed in rooms	1000 m	0.721 m	Ave LE, 98.73%
Acc, Gyro [80]	Assistive QR code with PDR	scan QR code along the path and kept smartphone in hand	35 m	N/A	Above 99%
IMU, BLE beacon [81]	BLE beacon, inertial dead reckoning	indoor environment	40 m	N/A	Above 97.47%
IMU, camera [82]	PDR, camera	meeting room	15 m	0.56 m	N/A
BLE-beacon [83]	Fuzzy logic, BLE fingerprinting	Indoor enviornment	25 m	0.43 m	N/A

**Table 2:** Description of notations and symbols used in the formulation.

Notation	Description
{inf}	Inertial navigational frame.
{sbf}	Sensor-body frame.
s∈R	Scaler part of quaternions.
v∈(x,y,z)	Vector part of quaternions, where $v\in R^{3}$.
*q*	Unit quaternion.
$q^{*}$	Conjugate quaternion.
⨂	Multiplication operation
$v^{inf}$	Vector in the inertial navigational frame.
$v^{sbf}$	Vector in the sensor body frame.
i,j, and *k*	A quaternion basis elements.
α,β,γ and δ	Quaternion real numbers.
$q_{inf}^{sbf}$	The unit-vector quaternion encoding rotation from the inertial navigational frame to the body frame of the sensor.
α	The amount of rotation that should be performed about the vector part.
$\sigma_{1}$, $\sigma_{2}$, and $\sigma_{3}$	Elements $\sigma_{1}$, $\sigma_{2}$, and $\sigma_{3}$ thought of as a vector about which rotation should be performed.
ϕ	The angle of rotation.
ϵ	Unit vector representing the axis of rotation.
Q(q)	Rotation matrix.
*Q*	Four-dimensional vector space over the real numbers $R^{4}$.
NED	North–east down
ψ	Rotation around yaw.
ξ	Rotation around pitch.
φ	Rotation around roll.
atan2	Computes the principal value of the argument function applied to the complex number in the quaternion.
δϕ	Prior gyros bias errors. The error between estimated gyroscope bias and true gyroscope bias.
δψ	Euler angles errors.
*x*	State vector of the proposed filter.
$e_{q}$	Error quaternions.
*e*	Attitude error.
${\dot{x}}_{k}=A_{k}x_{k}+Eu_{k}$	The state equation for the attitude estimation system.
$u_{k}$	The noise vector, which refers to the noise related to the rotation error angle.
$\zeta_{\delta \Psi }\left(t\right)$	Noise error, true bias random walk.
$\zeta_{\Delta \Phi }\left(t\right)$	Noise error, estimated bias random walk.
${\hat{w}}_{b/n^{\prime }}^{b}$	The estimated rotation rate.
${\tilde{acc}}^{sbf}$	Output of accelerometer.
${\tilde{mag}}^{sbf}$	Output of magnetometer.
*y*	Measurement of the combination of the accelerometer and magnetometer.
$\eta_{{\mathrm{acc}}^{sbf}}\mathrm{and}\eta_{{\mathrm{mag}}^{sbf}}$	The measurement independent zero-mean Gaussian white-noise.
$m^{n}$ and $g^{n}$	True magnetic and gravity vector.
$\eta_{acc^{sbf}}^{2}\;\mathrm{and}\;\eta_{mag^{sbf}}^{2}$	The variance of measurement noise.
$M_{R}$	Covariance matrix.
h(q)	Represents the nonlinear equations that convert the magnetometer reference vector $r_{mag}$ ∈ $R^{3}$ and accelerometer reference vector $r_{acc}$ ∈ $R^{3}$ from INF to the SBF.
ρ	Sigma points.
λ	Represents the scaling parameter that shows the sigma points spread around the column vectors of the covariance matrix.
$K_{x_{j}}^{\mathrm{prior}\;}$	The prior estimates of covariance.
ρ′	Posterior sigma points.
$W_{i}^{m}$ and $W_{i}^{j}$	Used to calculate the mean and covariance of the posterior sigma points.
μ	Determines the spread of the ρ around ${\hat{x}}_{k}^{\mathrm{prior}\;}$ and β accentuate the weighting on the zeroth ρ.
$\left(\tilde{y}\right)$	Residual error.
D(.)	Distribution constructed by the kernel density estimate.
*wO*,*t* (.)	Weight assigned to each activity performed in the various dedicated zones $Z_{i}$.

**Table 3:** Performed activities and range of accelerated values.

Performed Activities	Working on Computer	Running Activity	Walking Upstairs	Walking Activity	Writing on White Board
Range of Acc Values	$\alpha_{1}\leq \alpha_{o}\geq \alpha_{2}$	$\alpha_{2}\leq \alpha_{o}\geq \alpha_{3}$	$\alpha_{3}\leq \alpha_{o}\geq \alpha_{4}$	$\alpha_{4}\leq \alpha_{o}\geq \alpha_{5}$	$\alpha_{5}\leq \alpha_{o}\geq \alpha_{6}$

**Table 4:** Implementation environment.

Component	Description
BLE Beacon model	Estimote
CPU	32-bit ARM® Cortex M0
Power source	CR2477
Battery life	3 years
Ideal beacons range	70 m (230 feet)
Practical beacons range	30–40 m
Radio frequency	2.4 GHz UHF
Version	Bluetooth 4.0 Smart
Sensors embedded	Accelerometer, temperature

**Table 5:** Implementation environment.

Component	Description
Operating system	Android OS
Hardware	BLE-beacon ARM® Cortex®-M4 32-bit processor with FPU, Smartphone, Intel(R) Core(TM) i5-8500 CPU @ 3.00GH
Memory	DDR4-16GB RAM, 64 kB RAM
Libraries	Google API, Android Graph Library, Android Position Library
Front end framework	Swing based GUI
Core programming language	Java
IDE	Android Studio
Simulation time	60 s (1 min)

**Table 6:** Comparion in Position Error-Reference and EPBCM.

Reference Position	$X_{\mathrm{True}}$ Meters	$Y_{\mathrm{True}}$ Meters	$X_{\mathrm{EPBCM}}$ Meters	$Y_{\mathrm{EPBCM}}$ Meters	Position Error
Elevator Area	6	1.2	6.06	1.26	0.08
Conference Room	3	1.9	3.11	1.89	0.11
Stairs Area	10	2.4	10.32	2.36	0.32
Mobile Computing Lab	1	1	1.01	1.005	0.01
Networking Lab	15	3.74	15.65	3.66	0.65
Rest Area	20	5.32	20.11	5.4	0.14

**Table 7:** Comparison of reference position with EPBCM, PDR, and BLE-beacon-based localization algorithms.

Reference Position	$X_{\mathrm{True}}$ Meters	$Y_{\mathrm{True}}$ Meters	$X_{\mathrm{EPBCM}}$ Meters	$Y_{\mathrm{EPBCM}}$ Meters	$X_{\mathrm{BLE}-\mathrm{beacon}}$ Meters	$Y_{\mathrm{BLE}-\mathrm{beacon}}$ Meters	$X_{\mathrm{PDR}}$ Meters	$Y_{\mathrm{PDR}}$ Meters
Elevator Area	6	1.2	6.06	1.26	6.13	1.27	6.07	1.39
Conference Room	3	1.9	3.11	1.89	3.08	1.98	3.1	2.04
Stairs Area	10	2.4	10.32	2.36	10.7	2.47	11.3	2.54
Mobile Computing Lab	1	1	1.01	1.005	0.99	1.01	1.03	1.04
Networking Lab	15	3.74	15.65	3.66	15.09	3.33	16.2	3.64
Rest Area	20	5.32	20.11	5.4	21.4	5.53	21.89	5.62

## Data Availability

Not applicable.
